# News and Notes

**Published:** 1913-02

**Authors:** 


### NEWS AND NOTES

### AN ITALIAN REVIEW OF A GEORGIAN'S BOOK

It will be pleasing to the friends of Dr. Niles, as well as those who take an interest in Southern medical literature, to note this review, taken from a recent number of the *Policlinico,* a high grade medical journal, of Rome, Italy.

"PELLAGRA, by George M. Niles, W. B. Saunders Co., Philadelphia and London.

"It is a diligent treatise, which purports to be complete. It examines some new sides of the problem, and contains some new observations gathered in America; however, the work is made in a general way on the studies made in Italy.

"We will consider some chapters: The author recognizes that the etiologic problem of pellagra is still waiting solution, but he is much for "maidism," and throws light on some new doctrines, which he calls iconoclastic. In support of the pathogenic action of the corn, he cites some observations in which that action seems to be included.

'A woman observed by Bass had an abnormal appetite for the starch of corn (amylophagia).' Campbell demonstrated that many of the cheap candies sold in America are made with glucose obtained from corn.

"The author discusses the photodynamic doctrine of Raubitschek, the doctrine of Mizell, the doctrine of Sambon on the simulium reptans, and informs us of the negative experiences of transmission as made by Lavinder on Rhesus monkeys.

"The different schools, he observes, support their views with a zeal, which often becomes partizan, doing much harm to scientific progress.

"A valuable chapter is the one on the cure. The forms of cure are many, up to the 606, the transfusion of blood, appendicostomy, colostomy, the drainage of the gall-bladder, and many others, but very wisely the author insists upon medita-tion with arsenic and iodide of potassium, and the hygienic-dietetic cure.

"The author describes and portrays the first hospital for pellagrins in America; very elegant, charming, it looks like a little villa.

'All through the book we find a fine humor, which makes the reading of it pleasant and easy. It is well illustrated.

'To give an idea of the care which the author has put in details, we will say that he devotes an entire page to the dis-cusssion of the way to pronounce the word pellagra, and con-cludes by saying that the pronunciation should be acquired directly from the Italian."

---

### PHILIPPINE CIVIL SERVICE EXAMINATION.—ASSISTANT IN EXPERIMENTAL THERAPEUTICS (MALE).—MARCH 10, 1913

The United States Civil Service Commission announces an open competitive examination for assistant in experimental therapeutics, Philippine Service, for men only. From the register of eligibles resulting from this examination certification will be made to fill a vacancy in the position of research assistant in experimental therapeutics in the Bureau of Science, in Manilla, Philippine Islands, at a salary of $2,000 a year, and vacancies as they may occur requiring similar qualifications, unless it is found to be to the interest of the service to fill any vacancy by reinstatement, transfer or promotion.

This position offers to the ambitious capable physician a wide field for experimental therapeutic work. The Bureau of Science possess one of the largest and most favorably known research laboratories in existence and is in the immediate vicinity of the Philippine General Hospital, which is probably the best institution of its kind in the Eastern Hemisphere. In view of the excellent opportunities presented qualified persons are urged to enter this examination.

It will not be necessary for applicants to appear at any place for examination. Their eligibility will be determined upon the evidence furnished in connection with application and examination Form B. I. A. 2, concerning their training and the work which they have accomplished.

Applicants must be graduates in medicine, and in addition must show at least one year's postgraduate experience in conducting laboratory research work in experimental therapeutics, or, as equivalent to the year's work, they may submit copies of publications prepared by them, evidencing their ability to carry on original experimental therapeutics work. A person is desired who is especially qualified in research, and it is stated that, for one who is satisfactory, the prospects of promotion are excellent.

Statements as to training, experience, and fitness are accepted subject to verification.

Applicants must have reached their eighteenth but not their fortieth birthday on the date of the examination.

The medical certificate on Form B. I. A. 2 must be executed by some medical officer in the service of the United States. Applicants should appear before medical officers of the Army, Navy, Indian, or Public Health and Marine-Hospital service. If such officer can not be conveniently visited, a pension-examining surgeon may execute the certificate. Special arrangements have been made with pension-examining boards throughout the country to give such examination for a fee of $2, to be paid by the applicant. This certificate must not be executed by the family physician of the applicant. The medical officer should indicate his rank or official designation on such certificate. When it is impracticable, by reason of the applicant's distance from a Government physician or a pension-examining surgeon, to have the medical certificate executed by such physician, it may be executed by any reputable physician. Such person may be required to undergo another examination in case of appointment.

Each applicant will be required to submit with his application a photograph of himself, taken within three years, which will be filed with his papers as a means of identification. An unmounted photograph is preferred. The name and date of examination, the competitor's name, and the year in which the photograph was taken should be indicated.

This examination is open to all male citizens of the United States who comply with the requirements.

Special attention is invited to the favorable conditions in respect to transportation, leave of absence, clothing, etc., in this service, printed hereon.

Persons who comply with the requirements and desire this examination should at once apply for Form B. I. A 2 to the United States Civil Service Commission, Washington, D. C.; the secretary of the board of examiners, post office, Boston, Mass., Philadelphia, Pa., Atlanta, Ga., Cincinnati, Ohio, Chicago, Ill., St. Paul, Minnesota, Seattle, Washington, San Francisco, California; custom house, New Yor, N. Y., New Orleans, La., Honolulu, Hawaii; old customhouse, St. Louis, Mo.; or to the chairman of the Porto Rican Civil Service Commission, San Juan, P. R. No application will be accepted unless properly executed, including the medical certificate, and filed with the Commission at Washington prior to the hour of closing business on March 10, 1913. In applying for this examination the exact title as given at the head of this announcement should be used in the application. ISSUED FEBRUARY 6, 1913.

---

### ELIMINATION OF PAIN FOLLOWING INJECTIONS OF SALVARSAN

Injections of Salvarsan, while remarkably beneficial in effect, have nevertheless been a source of considerable agitation on account of the painful and other untoward symptoms which follow their use. The experimental mind, so prevalent among the medical profession, sought to connect these symptoms with the arsenical content in Salvarsan which undoubtedly must produce a sort of arsenical poisoning in the system. Scientific experiments along these lines, made by Dermathologists, proved these contentions and the treatment therefore by active catharsis by means of Hunyadi Janos Water has been found to eliminate pain and other constitutional symptoms instramuscular and intravenous injections of Salvarsan.

The technic, case reports and full results of these experiments have been published in an artistic booklet which we recommend, for perusal, to every physician.

The firm of Andreas Saxlehner, New York will, we are sure, glandly send you one upon request.

